# Immunostimulatory activity of inactivated environmental *Bacillus *isolates and their endospores

**DOI:** 10.1038/s41598-025-12833-7

**Published:** 2025-08-20

**Authors:** Max Dekeukeleire, Dieter Vandenheuvel, Tippapron Khondee, Lize Delanghe, Tim Van Rillaer, Sofie Thys, Jean-Pierre Timmermans, Sarah Lebeer, Irina Spacova

**Affiliations:** 1 Laboratory of Applied Microbiology and Biotechnology (LAMB), Department of Bioscience Engineering, University of Antwerp, Antwerp, Belgium; 2 Research group EnVOC (Environmental Organic Chemistry and Technology), Department of Green Chemistry and Technology, Faculty of Bioscience Engineering, Ghent University, Ghent, Belgium; 3 Laboratory of Cell Biology and Histology (CHB), Department of Pharmaceutical, Biomedical and Veterinary Sciences, University of Antwerp, Wilrijk, Belgium; 4 Antwerp Centre for Advanced Microscopy (ACAM), University of Antwerp, Wilrijk, Belgium

**Keywords:** Immunology, Microbiology, Bacterial host response

## Abstract

The spore-forming capacity of *Bacillus* spp. enables environmental persistence and stable product formulations, yet the interactions of environmental *Bacillus* spores and vegetative cells with the human immune system are not fully understood. We investigated the immunostimulatory potential of seven environmental *Bacillus* isolates (*B. subtilis*,* B. velezensis*,* B. licheniformis*,* B. pumilus*) and optimized their inactivation methods to preserve surface integrity and immunostimulatory activity. Inactivation was evaluated using heat/UV-C and 10–100% formalin, followed by scanning electron microscopy and human reporter cell assays (THP1-Dual monocytes, HEK-Blue hTLR2/6 and hTLR4). Heat/UV-C treatment retained *Bacillus* morphology and enhanced immune activation compared to 10–100% formalin treatment. Spores and vegetative cells activated key immune transcription factors (nuclear factor kappa B and interferon regulatory factors) in human monocytes, with spores inducing 1.5- to 8-fold lower responses compared to vegetative cells, and strain- and species-dependent effects observed. All tested *Bacillus* isolates significantly activated Toll-like receptor TLR2/6, but not TLR4. Genome analysis identified *Bacillus* spore envelope components (SpsAEIK, CotBCGQ) that may influence differences in immune responses. Our findings improve understanding of *Bacillus*-human immune interactions, revealing strain- and species-specific immunostimulatory activity. These results support further exploration of *Bacillus* isolates for immune-related and environmental applications.

**Supplementary Information:**

The online version contains supplementary material available at 10.1038/s41598-025-12833-7.

## Introduction

*Bacillus* species are aerobic, Gram-positive bacteria that are found in a wide range of environments, such as soil, vegetation, and the human and animal gastrointestinal tract^1–3^. The capacity of *Bacillus* species to form dormant and resistant structures, called spores, enables their survival in harsh environmental conditions, such as high temperatures, desiccation, ultraviolet radiation, and acidity^4,5^. The fact that spores can also survive production processes, transport, and storage, and are able to pass the highly acidic gastric environment and reach the intestines in a viable form, makes *Bacillus* strains interesting for many commercial and medical applications. Strains of *Bacillus subtilis*, *Bacillus licheniformis*, and *Bacillus pumilus* have been extensively explored for their potential beneficial capacities, such as biodegradation, anticancer, antioxidant, antimicrobial, and vitamin production properties, for the use in human health, agriculture, industry, bioremediation and biotechnological processes^3,4,6–11^.

Exposure to environmental microorganisms is proposed to have an important role for balanced immune development in the context of the Biodiversity hypothesis, which states that “contact with natural environments enriches the human microbiome, promotes immune balance and protects from allergy and inflammatory disorders“^12^. During environmental contact with *Bacillus* species or through the use of *Bacillus*-containing products, different human body sites can be exposed to vegetative bacteria and spores through inhalation, ingestion, and dermal contact. Several *Bacillus* species, including *Bacillus amyloliquefaciens*, *B. licheniformis*,* B. pumilus*,* B. subtilis*, and *B. velezensis*, are considered to be safe for humans and animals. These species are included in the qualified presumption of safety (QPS) list and many *Bacillus* strains are generally regarded as safe (GRAS) by the US Food and Drug Administration (FDA)^4,13^. Insights into the beneficial immunostimulatory capacity have been generated in vitro and in vivo for specific strains of *B. subtilis*,* B. pumilus*, and *B. coagulans*, but data are missing for dormant spores and other non-pathogenic *Bacillus* species^1,3,14–18^.

The immunostimulatory effects of beneficial bacteria are generally mediated through pattern recognition receptors (PRRs), such as Toll-like receptors (TLRs), which recognize bacterial ligands or microbe-associated molecular patterns (MAMPs)^19^. Previous studies have shown that model probiotics such as *Lacticaseibacillus rhamnosus* GG and *Escherichia coli* Nissle 1917 in sufficient concentrations can exert their beneficial effects on the host via TLR2/6 and TLR4, respectively^20,21^. These MAMP-PRR interactions result in the activation of downstream immune transcription factors, such as the nuclear factor kappa-light-chain-enhancer of activated B cells (NF-κB) that initiates the transcription of cytokine and chemokine genes and regulates activation and differentiation of immune cells, and interferon regulatory factors (IRF) that regulate transcription of interferon-related genes^22,23^. While interactions with TLRs and induction of NF-κB by *B. anthracis* and *B. subtilis* and their spores have previously been shown^1,17,18^, the studied *Bacillus* species are limited. Importantly, none of the previous studies have inactivated the vegetative bacteria and spores with preservation of surface molecules^24^. Since the spores investigated in these studies were still capable of germination and outgrowth, the question remains whether the immune interactions are triggered primarily by the initial interaction with surface molecules on dormant spores or by molecules that become available after spore germination and outgrowth.

The cell wall in vegetative cells mainly comprises a thick peptidoglycan layer, recognized by TLRs and Peptidoglycan Recognition Proteins^25–27^. Although poorly studied in *Bacillus*, immunological responses are often further influenced by exopolymeric substances (EPS) surrounding the peptidoglycan layer^28,29^. The understanding of the function and chemical composition of the EPS is, however, relatively limited in *Bacillus* strains^28^. On the other hand, the spore envelope is well-documented and consists of peptidoglycans, forming the cortex, and large structural (glyco)proteins, forming a spore coat^30,31^. Some species, including *Bacillus anthracis* and *B. cereus*, contain an additional layer surrounding the spore coat, called the exosporium. The spore coat, consisting of the inner coat, outer coat, and crust, can vary considerably both in composition and in thickness between species^31,32^. More specifically, species can have different amounts of coat layers with varying coat layer arrangements, individual coat proteins, and appendages extending from the coat surface^31^. Genome sequence analysis reveals that roughly half of the known *B. subtilis* coat protein genes have identifiable orthologues in other *Bacillus* species, while the remaining half seem to lack conservation entirely^32^. These spore coat proteins can interact with the environment or host organism, but detailed research on the immunological interaction of spores and the human immune system is lacking^32,33^. Further research is also needed to differentiate the immunostimulatory effects of surface molecules on *Bacillus* spores and vegetative cells.

 In this study, we investigated the immunostimulatory effects of environmental *B. velezensis*, *B. licheniformis*, *B. subtilis*, and *B. pumilus* isolates from the soil (Table 1), both as spores and as vegetative cells. We specifically compared the immunostimulatory capacity of inactivated *Bacillus* spores to their inactivated vegetative form. While most inactivation protocols described for bacilli focus on their destruction (e.g., for *B. anthracis*^34^ and the food industry^2^, we focused on inactivation methods that can preserve the extracellular microbial structures. Inactivation using ultraviolet (UV) radiation, heat, and formalin was optimized for the treatment of spores and vegetative cells, with the preservation of the integrity of the cell surface in mind. To understand the immunological impact of inactivated vegetative and sporulated *Bacillus* isolates on human reporter cell lines, we assessed the induction of TLR4 and TLR2/6 immune receptors as key for bacteria-host interactions, and the activation of the NF-κB and IRF transcription factors important for innate and adaptive immunity. By focusing on the induction of key transcription factors and evaluating different inactivation methods, we aimed to advance the understanding of the role that environmental *Bacillus* isolates and their spores might play for human immune health.

## Results

### Heat/UV-C combination or concentrated formalin treatment achieves complete inactivation of *Bacillus* spores and vegetative cells

As a starting point, the efficacy of several inactivation methods applied to spores and vegetative cells of environmental *Bacillus* isolates was investigated, which was instrumental in distinguishing the immunostimulatory capacity of spores versus vegetative cells in subsequent cellular assays. We aimed to target a range of *Bacillus* species, as efficacy of inactivation methods may vary between bacterial species. Inactivation requirements were determined using a panel of *Bacillus* strains, as representative for the *Bacillus* species used in the study, which included *B. velezensis* AMBY1, *B. licheniformis* AMBY4, *B. subtilis* AMBY8, and *B. pumilus* AMBY11. The goal was to inactivate vegetative cells to prevent overgrowth during human cell experiments and inactivate spores to minimize germination, while preserving the cell surface microbial structures responsible for microbe-host interactions^35,36^. Physical inactivation by heat, UV-C irradiation, and chemical inactivation using ethanol or formalin were selected because of their feasibility and common use as inactivation techniques for gram-positive bacteria^35–38^. Furthermore, a combination of some of these techniques was used to test for additive effectiveness^5^.

First, heat inactivation (50–100 °C for 15–40 min) was tested on vegetative cells and spores of selected strains representative of four *Bacillus* species. All tested vegetative *Bacillus* cells survived heat exposure to 50 °C, but not 100 °C (Supplementary Table S1). Heat treatment between 60 and 80 °C was able to inactivate vegetative cells, but was insufficient to inactivate *Bacillus* spores. Next, UV-C exposure was tested and led to complete inactivation of vegetative cells of *B. subtilis* and *B. velezensis* after 3 h, while for *B. licheniformis* and *B. pumilus* a few colonies still grew after 4 h (Supplementary Table S1). When a purified spore suspension of *B. velezensis* AMBY1 and *B. pumilus* AMBY11 was exposed to UV-C, complete inactivation was observed after 10 min. Treatment with 10 and 100% ethanol at 20 °C did not lead to complete inactivation of the tested bacilli (Supplementary Table S1). Finally, 0.5% and 1% formalin had no effect on the vegetative *Bacillus* viability based on CFU counts, while complete inactivation was achieved with 10% formalin for 1 h and 100% formalin for 5 and 10 min (Supplementary Table S1). As heat inactivation was only observed for vegetative *Bacillus* cells and UV-C inactivation was effective against *Bacillus* spores, the combination of heat followed by UV-C irradiation was tested on vegetative and sporulated *B. velezensis* AMBY1, *B. licheniformis* AMBY4, *B. subtilis* AMBY8, and *B. pumilus* AMBY11 (Table 1). Complete inactivation of both spores and vegetative cells of the four *Bacillus* strains was achieved by 70 °C heat followed by 2 h UV-C exposure (2.5 h UV-C for *B. velezensis* AMBY1), or 60 °C heat followed by 3 h UV-C exposure (with nearly complete inactivation for *B. velezensis* AMBY1 resulting in only 1 non-inactivated colony observed). Taken together, the optimal inactivation efficacy for both spores and vegetative cells of all tested *Bacillus* species was observed with either 10% formalin, or with heat treatment of 60 °C for 20 min followed by 3 h of UV-C treatment.

**Table 1:** Colony counts after heat inactivation followed by UV-C inactivation of vegetative *Bacillus* spp. And their spores starting from an inoculum of 2 × 10^6^ CFU/ml. Results are representative of 2 repeats, and data is presented as mean ± sd of three technical replicates of 10 µL of plated-out inactivated culture.

Heat/UV-C inactivation													
UV inactivation time		2 h				2.5 h				3 h			
Temperature		60 °C		70 °C		60 °C		70 °C		60 °C		70 °C	
**Heat inactivation time**		**20 min**	**30 min**	**20 min**	**30 min**	**20 min**	**30 min**	**20 min**	**30 min**	**20 min**	**30 min**	**20 min**	**30 min**
***Bacillus velezensis*** ** AMBY1**	Vegetative	3.3 ± 0.6	4.3 ± 2.5	10.3 ± 2.9	11.0 ± 1.7	1.0 ± 1.0	0.3 ± 0.6	0.0	0.0	0.0	0.3 ± 0.6	0.0	0.0
	Spore	0.0	0.0	0.0	0.0	0.0	0.0	0.0	0.0	0.0	0.0	0.0	0.0
***Bacillus licheniformis*** ** AMBY4**	Vegetative	0.7 ± 1.2	0.0	0.0	0.0	0.0	0.3 ± 0.6	0.7 ± 1.2	0.0	0.0	0.0	0.0	0.0
	Spore	0.0	0.0	0.0	0.0	0.0	0.0	0.0	0.0	0.0	0.0	0.0	0.0
***Bacillus subtilus*** ** AMBY8**	Vegetative	6.0 ± 2.0	2.7 ± 0.6	0.0	0.0	2.0 ± 0	1.7 ± 1.2	0.0	0.0	0.0	0.0	0.0	0.0
	Spore	0.0	0.0	0.0	0.0	0.0	0.3 ± 0.6	0.0	0.0	0.0	0.0	0.0	0.0
***Bacillus pumilus*** ** AMBY11**	Vegetative	0.3 ± 0.6	0.0	0.0	0.0	0.0	0.0	0.0	0.0	0.0	0.0	0.0	0.0
	Spore	0.0	0.0	0.0	0.0	0.0	0.0	0.0	0.0	0.0	0.0	0.0	0.0

To ensure immunostimulatory activity after inactivation, it was important to retain intact membranes and surface proteins of *Bacillus* cells. Potential damage to the inner membrane was assessed by quantifying the release of nucleic acids and proteins from spores after heat/UV-C treatment, as described by Li et al.^39^. Based on absorbance measurements, no significant increase in nucleic acid and protein concentration was detected in the bacterial suspension (Supplementary Table S2).

### Heat/UV-C- or formalin-treated *Bacillus* cells and spores do not demonstrate major changes in cell morphology

**Fig. 1:**
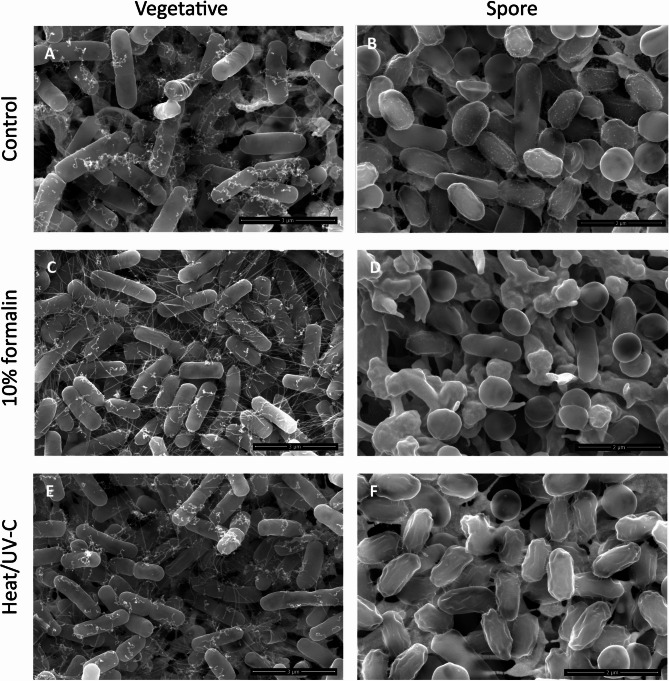
Effects of formalin and heat/UV-C inactivation on cell morphology of vegetative (A, C, E) and sporulated (B, D, F) *B. pumilus* AMBY11 visualized by scanning electron microscopy (SEM). Scale bar = 2 μm (spores) and 3 μm (vegetative cells). (A-B) untreated control cells, (C-D) cells inactivated by 10% formalin, (E-F) cells inactivated by heat followed by UV-C.

Next, we aimed to visualize vegetative *Bacillus* cells and their spores after inactivation by 10% formalin, or heat/UV-C, to understand the potential damaging effects of these treatments on the cell integrity and surface morphology. *B. pumilus* AMBY11 was selected as a representative strain. Scanning electron microscopy (SEM) of untreated vegetative *B. pumilus* AMBY11 cells (Fig. 1A) revealed a rod-shaped morphology with the presence of pili or fimbriae-like structures. Heat/UV-C treatment or 10% formalin did not cause any dents or cell ruptures in the vegetative *B. pumilus* AMBY11 cells. Pili or fimbriae appeared less curly after inactivation by 10% formalin compared to the control and heat/UV-C inactivated samples.

Untreated spores of *Bacillus* AMBY11 were generally oval-shaped with a smooth or shriveled surface (85%), with a small percentage (15%) presenting a rather spherical smooth-surfaced morphology, often containing small protrusions. Treated spores (either formalin or heat/UV-C) did not show any cell ruptures or dents, indicating minimal spore coat damage. Upon treatment of spores, some morphological changes become noticeable. The formalin-treated spores were mostly spherical shaped with a smooth surface (80%), while the heat/UV-C-treated spores were mainly oval-shaped with a shriveled surface (71%) and showed less protrusions in comparison with the untreated spores.

### Immunostimulatory capacity of vegetative *Bacillus* isolates after heat/UV-C inactivation is superior to formalin inactivation

**Fig. 2:**
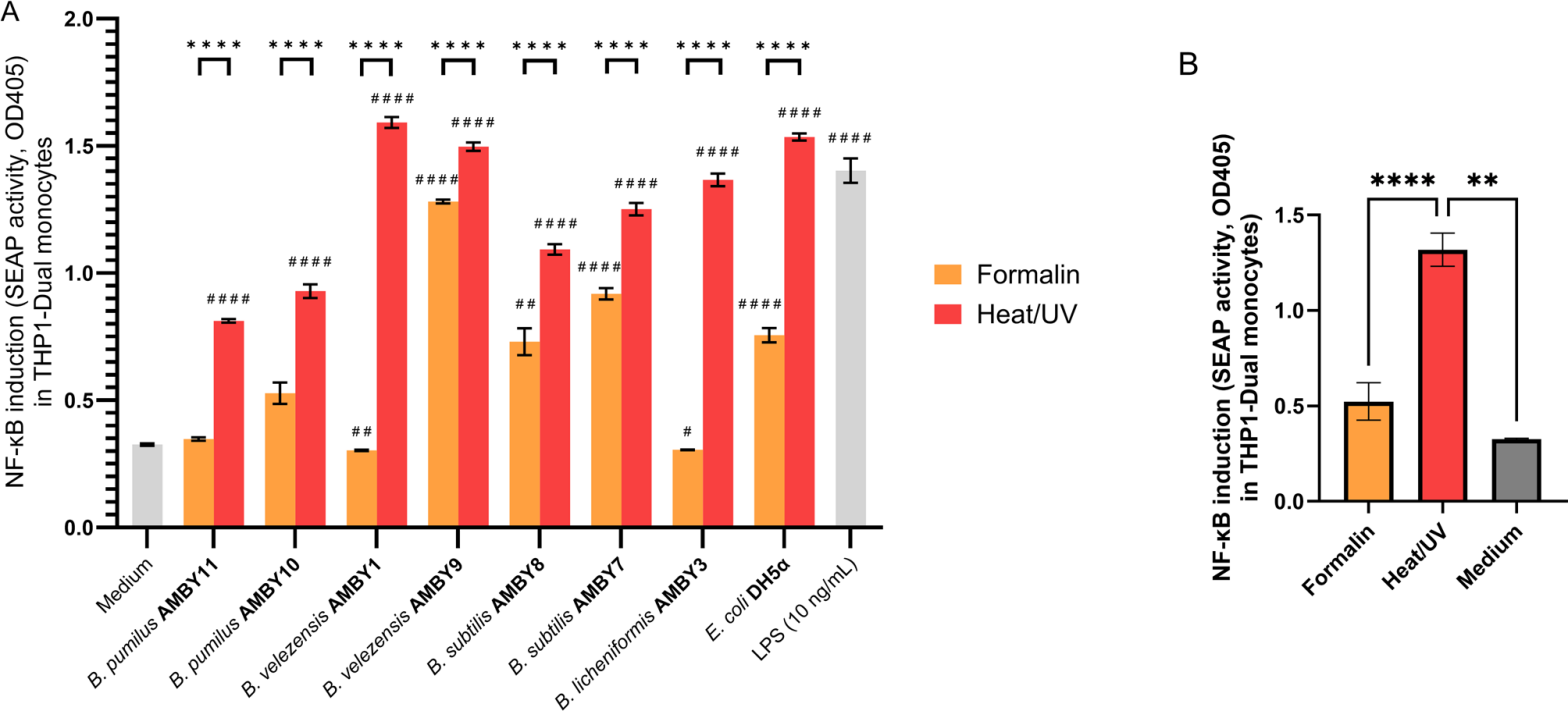
NF-κB immune transcription factor induction capacity of vegetative *Bacillus* cells in human THP1-Dual™ monocytes after inactivation with formalin compared to heat/UV-C. Results are shown per strain (A) or as an average of all *Bacillus* strains combined (B). Bars depict means ± SEM. One-way ANOVA test, followed by Dunnett’s multiple comparison test, was used to test differences between heat/UV and formalin within the same strain, and between conditions compared to the medium control. Significant differences between treatments are depicted by *p < 0.05, **p < 0.01, ***p < 0.001, and ****p < 0.0001. Significant difference compared to the medium control is indicated by #*p* < 0.05, ##*p* < 0.01, ###*p* < 0.001, and ####*p* < 0.0001. LPS at 10 ng/mL was used as positive control.

After determining the impact of heat/UV-C and formalin inactivation treatments on the *Bacillus* cell morphology, we assessed whether potential modifications in cell surface molecules due to inactivation impacted the immunostimulatory interactions of seven environmental *Bacillus* strains with host immune cells, namely *Bacillus velezensis* AMBY1, *Bacillus velezensis* AMBY9, *Bacillus licheniformis* AMBY3, *Bacillus subtilis* AMBY7, *Bacillus subtilis* AMBY8, *Bacillus pumilus* AMBY10, and *Bacillus pumilus* AMBY11. To this end, THP1-Dual™ human reporter monocytes were exposed to vegetative formalin- or heat/UV-C inactivated *Bacillus* strains, and the induction of the key immune transcription factor nuclear factor kappa-light-chain-enhancer of activated B cells (NF-κB), which regulates multiple aspects of innate and adaptive immunity, was evaluated (Fig. 2). All heat/UV-C-inactivated *Bacillus* spp. and the non-pathogenic control laboratory strain of *E. coli* DH5α significantly induced NF-κB. Strains belonging to the same species led to similar NF-κB induction, with no significant differences between *B. pumilus* AMBY10 and AMBY11 (*p* = 0.9994), *B. velezensis* AMBY1 and AMBY9 (*p* = 0.0773), or *B. subtilis* AMBY7 and AMBY8 (*p* = 0.9961). On the contrary, a significantly lower read-out was observed for formalin-treated bacteria compared to heat/UV-C-inactivated bacteria for all tested strains. Furthermore, inactivation with 10% formalin resulted in non-significant induction or absence of NF-κB induction by vegetative *B. pumilus* AMBY11, *B. velezensis* AMBY1, and *B. licheniformis* AMBY3 compared to exposure to cell medium without bacteria. When readouts of all tested *Bacillus* strains were combined based on inactivation condition, the statistical analysis demonstrated that NF-κB induction in human monocytes by formalin-inactivated *Bacillus* strains was not significantly higher (*p* = 0.6757) compared to NF-κB induction by medium without bacteria (Fig. 2B). Although heat/UV-C inactivation may induce a slightly more negative effect on the morphology of the vegetative cells and spores (Fig. 1), chemical fixation using formalin resulted in a significantly greater impact on immunostimulatory capacity, as shown by the cell culture assay. This led to the conclusion that formalin inactivation of *Bacillus* strains was therefore a less suitable method in the context of immunostimulation.

### Inactivated *Bacillus* isolates demonstrate IRF and NF-κB induction capacity which differs between spores and vegetative cells

**Fig. 3:**
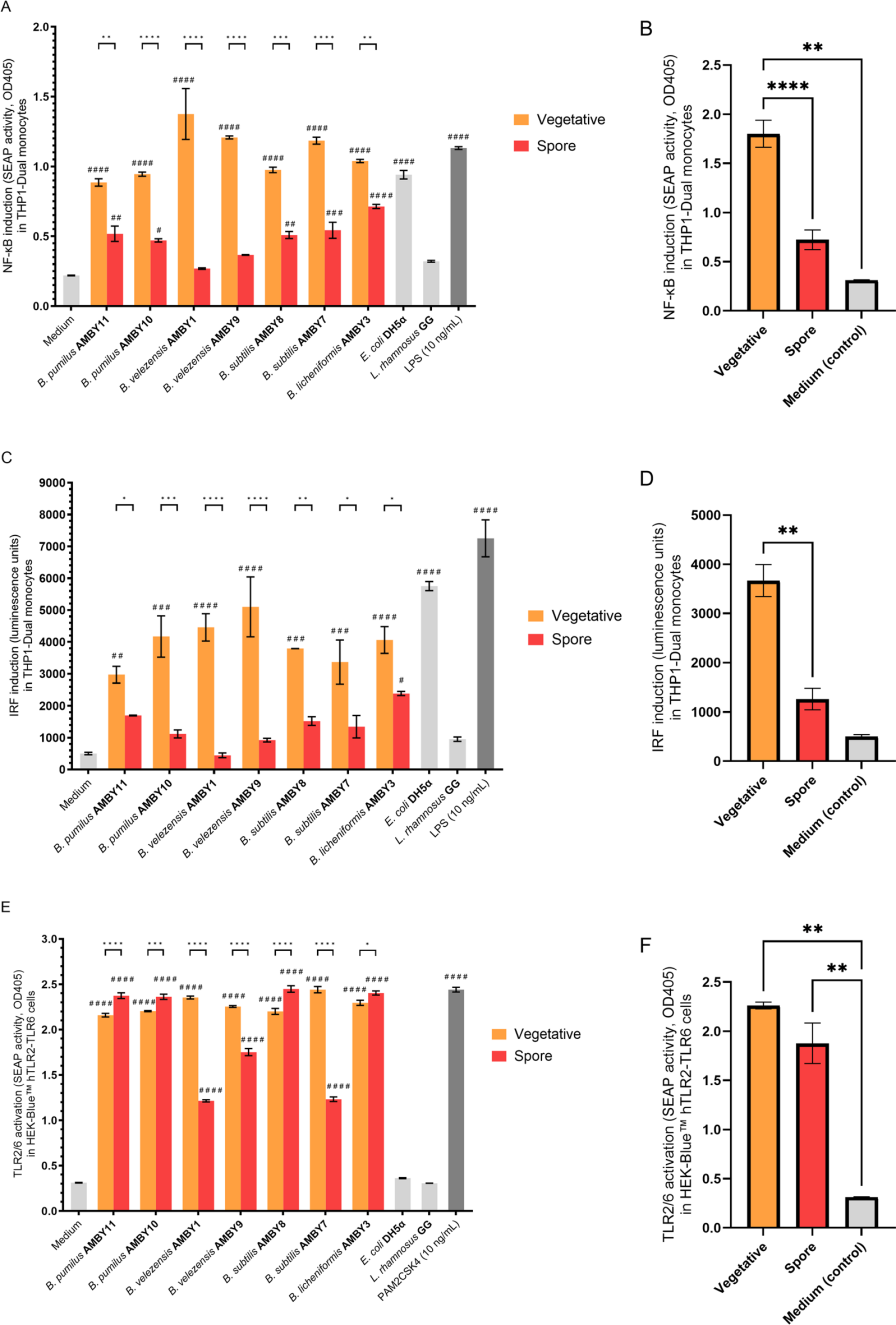
Induction capacity of inactivated vegetative *Bacillus* spp. compared to inactivated spores for (A, B) nuclear factor κB (NF-κB) and (C, D) interferon regulatory factors (IRFs) in human THP1-Dual™ monocytes, and (E, F) TLR2/6 activation in HEK-Blue™ cells. (B, D, F) Combined average induction of NF-κB (B), IRF (D), and TLR2/6 (F) by spores and vegetative cells. The medium condition represents unexposed cells and serves as a baseline, while lipopolysaccharide (LPS) and PAM2CSK4 at 10 ng/ml serve as immune activation controls, and *E. coli* DH5α and *L. rhamnosus* GG serve as non-pathogenic and probiotic controls, respectively. Bars depict means ± SEM. Corrected significance determined by one-way ANOVA, followed by Dunnett’s multiple comparison test compared between spores and vegetative cells (*p < 0.05, **p < 0.01, ***p < 0.001, and ****p < 0.0001). Difference compared to the medium control is depicted by #*p* < 0.05, ##*p* < 0.01, ###*p* < 0.001, and ####*p* < 0.0001.

Once we determined that heat/UV-C inactivation of the tested *Bacillus* isolates was superior regarding the retention of immunostimulatory activity, we used this inactivation method to determine the ability of inactivated *Bacillus spp.* and their spores to stimulate key immune pathways in human cells. Inactivation allowed us to estimate the immunostimulatory properties of spores and minimalizing the subsequent effects resulting from germination and outgrowth; conversely, inactivation of vegetative cells prevented their potential sporulation. All purified spore suspensions were visualized with confocal microscopy after a Schaeffer–Fulton stain before adding them to human cells (Supplementary Figure S1). We have focused on the induction of IRF and NF-κB as key immune transcription factors necessary for antiviral and other general innate immune system responses. The tested strains induced similar patterns between IRF and NF-κB read-outs relative to each other (Fig. 3), with more closely related *Bacillus* strains showing similar immunostimulatory capacity. The vegetative bacilli significantly activated the IRF and NF-κB pathways in human monocytes compared to the cell medium without bacteria. Of all tested bacilli, *B. velezensis* AMBY1 and AMBY9 were the strongest IRF inducers with more than nine-fold induction compared to the medium control and more than a four-fold induction compared to the model probiotic *L. rhamnosus* GG. Vegetative *B. subtilis* AMBY7, *B. velezensis* AMBY1 and AMBY9 also led to the strongest NF-κB induction among bacilli with approximately an eightfold and fivefold higher induction compared to the medium control and *L. rhamnosus* GG, respectively. On the contrary, no significant activation of the IRF pathway was observed following exposure to inactivated spores, with the exception of *B. licheniformis* AMBY3, although a trend towards activation was noted for most of the bacilli. Aside from *B. velezensis*, spores were capable of inducing a significant NF-κB response when compared to medium control, however with a lower induction capacity than that observed for vegetative *Bacillus* cells. When combining readouts based on metabolic state, statistical analysis revealed that spores did not elicit a significantly higher NF-κB response (*p* = 0.5053) compared to unexposed cells (Fig. 3B). Overall, spores induced a significantly lower activation of both NF-κB and IRF pathways compared to vegetative cells (Fig. 3B and D).

### Spores and vegetative cells of *Bacillus* isolates activate TLR2/6 but not TLR4

Next, we investigated which TLR receptors contributed to the observed NF-κB and IRF activation by sporulated and vegetative *Bacillus*. We focused on TLR2/6 and TLR4, as these are key TLR receptors involved in the immunostimulatory action of many beneficial bacteria, and the induction of TLR2/6 has previously been reported in murine macrophages by vegetative gut *Bacillus* isolates^18, ^but is less studied than environmental *Bacillus* isolates. When co-cultured with HEK-Blue™ reporter cells, both sporulated and vegetative *Bacillus* spp. were recognized by human TLR2/6 resulting in a seven-fold activation by vegetative cells and six-fold activation by spores compared to the medium controls (Fig. 3E and F). Between the spores, the lowest activation of TLR2/6 was observed in *B. subtilis* AMBY7, and *B. velezensis* AMBY1 and AMBY9. On the contrary, no activation of TLR4 was observed by vegetative cells or spores of the tested bacilli compared to the medium control, as well as no cytotoxicity (Supplementary Figures S2 and S3).

### Comparative genomic analysis of wall structure and surface properties of environmental *Bacillus* strains

**Fig. 4:**
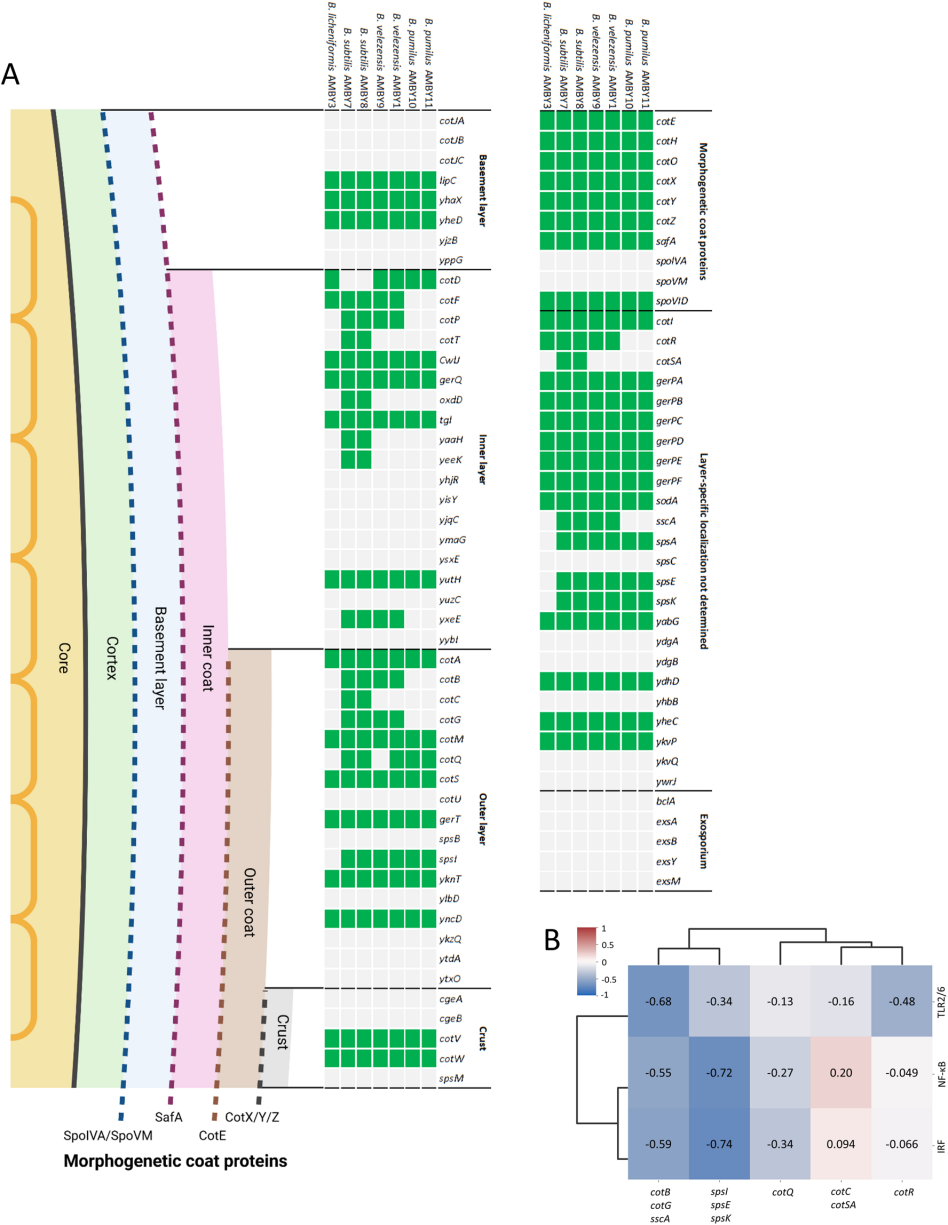
(A) Homologous spore coat protein sequences annotated in the tested *Bacillus* spp. (in green: detected, in grey: not detected with BLASTp). The core is protected by the cortex and the spore coat. The spore coat consists of four layers: the basement layer (blue), the inner coat (purple), the outer coat (brown), and the crust (grey). (B) Pearson correlation between normalized immune cell culture readouts (TLR2/6, NF-κB, IRF) and gene profiles of genes related to outer spore layer, crust, and undetermined localization. Pearson correlation coefficients are depicted in color [scale from blue (−1) to red (1)] and as numbers. Genes present or absent in all strains were not included.

To better understand the species- and strain-specific differences between the tested *Bacillus* isolates regarding their cell surface molecules capable of driving immune interactions with host cells, we have conducted a genome analysis for the presence of sequences of various trace coat proteins or carbohydrates in the outer coat and crust of the tested *Bacillus* isolates (Fig. 4A)^32,33^. In total, 53 homologous protein sequences were found in the *B. subtilis* AMBY7 and AMBY8 genomes related to the spore coat structure. From these, the sequences for *cotC*, *cotSA*, *cotT*,* oxdD*,* yaaH*, and *yeeK* were not found in *B. pumilus* AMBY10 and AMBY11, *B. velezensis* AMBY1 and AMBY9, or *B. licheniformis* AMBY3. Additionally, *cotB*, *cotG*, *cotP*, *sscA*, and *yxeE* were also not annotated in *B. licheniformis* AMBY3 and *B. pumilus* strains. Spore coat proteins *cotF* and *cotR* were not annotated in *B. pumilus* AMBY10 and AMBY11, and *spsA*, *spsE*, *spsI*, and *spsK* homologs were not found in *B. licheniformis* AMBY3. The *cotQ* gene was not annotated in the genome of *B. velezensis* AMBY9 and *B. licheniformis* AMBY3 compared to the other genomes. Finally, *cotD* was found in the genome of *B. pumilus* AMBY10 and AMBY11, *B. velezensis* AMBY1 and AMBY9, and *B. licheniformis* AMBY3, however not in the genome of *B. subtilis* strains. *SscA* and *cot* genes encode the structural components of the spore coat and *oxdD* is responsible for post-translational modifications^40,41^. *Sps* genes are involved in the synthesis of polysaccharides (PS) located on the spore surface and play a crucial role in crust structure and spore surface properties, influencing spore surface hydrophilicity and adhesion capacity^42,43^. *YaaH* encodes peptidoglycan hydrolases, which play a minor role in the degradation of the cortex peptidoglycan, while the function of *yeeK* and *yxeE* are not yet identified in literature^32^. Presence of the *spsE*, *spsI*, and *spsK* genes was negatively correlated with the induction of NF-κB (−0.72, *p* = 0.45) and IRF (−0.74, *p* = 0.45) pathways by spores based on Pearson correlation (Fig. 4B). C*otB*, *cotG* and *sscA* were found to be negatively correlated with TLR2/6 activation (−0.68, *p* = 0.45) and induction of IRF (−0.59, *p* = 0.59) and NF-κB (−0.55, *p* = 0.59) pathways by spores. Regarding putative MAMPs of vegetative bacilli cells, a difference in the presence or absence of genes between strains was also observed related to the production of structural exopolymeric substances (encoded by *epsO*, *sacB*, and *sacC*), surface active proteins (encoded by *fenF* and *bamB*), and enzymes (encoded by *aprE*, *mpr*, *nprB*, *nprE*, and *pel*), while annotated genes related to extracellular flagellar proteins were similar in all genomes (Supplementary Table S3).

## Discussion

Environmental *Bacillus* species are frequently found in habitats to which humans are exposed. While specific *Bacillus* strains and their spores are increasingly explored for health-promoting applications, these applications require better characterization of immunostimulatory capacity of *Bacillus* isolates, and particularly the immunostimulatory effects of *Bacillus* vegetative cells versus spores as such are not well understood. The goal of this study was to investigate the immunostimulatory capacities of spores and vegetative cells of environmental *B. licheniformis*,* B. pumilus*,* B. subtilis*, and *B. velezensis* soil isolates using an optimized inactivation methodology.

To assess the impact by spores on human cells with minimal germination or outgrowth, or by vegetative cells without the impact of overgrowth, heat/UV exposure was established as a suitable inactivation method with a focus on maintaining the *Bacillus* cellular integrity and morphology^37^. We achieved complete spore and vegetative cell inactivation for different environmental strains of *Bacillus* by 10% formalin treatment for 1 h at 20 °C or by combining 60 °C heat for 20 min followed by 3 h of UV-C radiation (5.40 J/cm²). Combining heat and UV-C was necessary, as 60 to 70 °C heat alone did not inactivate spores of *Bacillus * spp., confirming previous reports^44^. Some morphological changes were observed using SEM for the *B. pumilus* AMBY11 spores after UV/heat or formaldehyde exposure. While the SEM images were not quantitative, they have provided a preliminary indication of shrinkage and wrinkling of spores under the influences of heat/UV-C compared to the untreated spores. This is in line with the observations in other papers by Zeng, et al.^45^ and Öberg, et al.^46^, where UV exposure resulted in wrinkling and shrinking of spores and was linked to the release of small amount of intracellular contents. Heat exposure to 80 °C was also associated with a slight visible shrinkage with little effect on the morphological characteristics of the *B. cereus* spores by Lv, et al.^47^. We observed some heterogeneity in spore morphologies, and especially formaldehyde-treated spores showed an increase in rounded morphologies and smoothing of the surface. This could be caused by several factors, including specific previous sporulation conditions, potential limited germination, or artifacts by formalin and/or SEM fixation effects. For example, specimens of *Bacillus thuringiensis* spores can naturally exhibit different shapes due to parasporal crystal formation, while post-sporulation treatments (e.g. for fluorescence in situ hybridization) have been reported to cause *Bacillus* spores to appear more spherical or collapsed^48,49^. The sporulation quality may also be influenced by the employed sporulation method. Sporulation using LB agar plates has been shown to result in a more defective coat structure, as evidenced by reduced resistance and altered germination behavior compared to spores generated on double-strength Schaeffer’s-glucose (2×SG) plates^50^. More homogenous treatments could be acquired by other sporulation methods, such as Difco sporulation media, 2×SG, and Mn²⁺-amended 10% Columbia Broth, which have been recommended in other studies, although their sporulation efficiency were reported to be species-dependent^51^. Heat/UV exposure was selected for inactivation purposes as no major morphological changes, no dents or ruptures, and minimal release of DNA were observed.

UV-C irradiation was effective for purified spore suspension to prevent colony formation, but required longer exposure times for vegetative cultures. Presumably, inactivation rates of vegetative cells are influenced by the medium’s composition. Spores were inactivated in ddH_2_O, while vegetative cells were inactivated in RPMI, characterized by a relative higher optical density^52^. UV-C inactivation has been explored in previous research using a comparable setup, where 90% inactivation of *B. thuringiensis* suspended in a petri dish was observed after 7.5 to 9 min exposure to 0.6 mW/cm², however 100% inactivation was not investigated^53^. We have explored the combination of UV-C radiation followed by heat for the first time in the tested *Bacillus* species. Gayán, et al.^52^. investigated a more complicated UV and heat method on *B. coagulans spores* by combining a higher UV-C radiation (27.10 J/mL) by a custom made UV reactor, with a shorter mild heat treatment (60 °C for 3.58 min) on spores. Compared to Gayán, et al.^52^. our protocol was successful using a laboratory-grade UV lamp for inactivation in Eppendorf tubes with preserved integrity of both spores and vegetative cells. While our protocol was successful, future experiments should explore the possibility of first implementing UV-C followed by heat in the *Bacillus* isolates tested here to potentially reduce the inactivation time.

An important goal of our study was to understand the impact of inactivation on the immunostimulatory capacity of the environmental *Bacillus* isolates. In our study, we confirmed the sporicidal activity of 10% formalin^54^, however NF-κB immune transcription factor induction was diminished in human monocytes exposed to *Bacillus* spp. inactivated by formalin compared to heat/UV-C. As no gross cell damage was observed with SEM, this suggests that the reduction in immunostimulatory activity due to formalin treatment is rather a result of ethoxylated adducts, crosslinks, depurination fragments, and hydrophobic inversions^55^. In other studies, *E. coli* O157:H7 and *Clostridium chauvoei* inactivated with much lower concentrations of formalin (0.4–0.5%) induced significant antibody response in rabbit and mouse models^56,57^ even higher compared to heat-inactivated cells by *E. coli* O157:H7^57^. Lower formalin concentrations may be better at preserving MAMPs^55^, however, lower concentrations were not sufficient to inactivate the environmental *Bacillus* strains tested in our study, potentially rendering formalin inactivation unreliable for *Bacillus* microbe-host interactions.

Based on retention of immunostimulatory activity and minor morphological alterations, heat/UV-C treatment was used to investigate the immune-inductive potential of environmental *Bacillus* isolates. Vegetative cells of all the *B. licheniformis*,* B. pumilus*, * B. subtilis*, and *B. velezensis* strains tested were able to significantly activate the key immune transcription factors NF-κB and IRF in human monocytes. To our knowledge, among the tested *Bacillus* species, induction of NF-κB has only been investigated for *B. subtilis*, where pretreatment led to suppression of NF-κB-mediated inflammation^16,58^. Potential induction of IRF pathways in immune cells (e.g., macrophages) has so far only been investigated in one study by non-inactivated *B. anthracis* and *B. subtilis* spores, where spores of another *B. subtilis* strain did not induce a strong expression of interferon-stimulated genes (ISG)^18^. In vivo studies in rabbits, broilers, and mice fed with *B. subtilis* or *B. licheniformis* have suggested that immunostimulatory activity with an increase in host cytokine production such as interleukin (IL)−6 and the regulatory IL-10 can be linked to improved immunity and bacterial infection resistance^1,59,60^. We showed that the induction capacity of NF-κB and IRF pathways was strain-dependent, and *B. velezensis* strains exhibited the strongest induction of NF-κB and IRF among the tested isolates, thus representing potential immunostimulatory probiotic candidates for in vivo testing. Our previous study on the development of an antiviral biotherapeutic throat spray demonstrated that model probiotic lactobacilli such as *L. rhamnosus* GG and *Lactiplantibacillus plantarum* WCFS1 are likewise potent activators of NF-κB and IRF in human monocytes^23^, as well as TLR2/6^20^. The tested environmental *Bacillus* strains exhibited a notably higher activation of NF-κB, IRF, and TLR2/6 compared to the model probiotic *L. rhamnosus* GG, which was less potent in our study likely due to the lower bacterial concentrations compared to those tested by Spacova et al.^23^ and Claes et al.^20^, which highlights the concentration-dependent nature of immune stimulation by probiotic and other bacteria, as previously demonstrated by Claes et al.^20^. Consequently, *Bacillus* species potentially represent one type of beneficial environmental bacteria that can interact with the human immune system in the context of the biodiversity hypothesis^12^.

The spores of all the *B. licheniformis*,* B. pumilus*,* B. subtilis*, and *B. velezensis* strains tested in our study were able to induce NF-κB and IRF pathways, but to a lesser extent compared to vegetative cells. The reduced expression is in accordance with Choo et al.^18^, where *B. subtilis* spores were not able to express ISG in mouse macrophages, while for other tested species this has not been reported yet. Of note, all tested *Bacillus* strains and their spores were capable of significant TLR2/6 (but not TLR4) induction, indicating that diminished IRF induction by spores or the differences between strains could not be due to less TLR2/6 activation. Our data on *B. velezensis*, *B. pumilus*, *B. licheniformis*, and *B. subtilis* complements other studies which mainly focused on the interaction of *B. subtilis* spores with dendritic cells and macrophages via TLR2 and promoting their maturation and enhancing adaptive immune responses^1,63^. It should be noted that some of the spore preparations in the present study contained intact vegetative cells at 0–1% (Supplementary Figure S1), and traces of cell debris not readily detectable with light microscopy were visible in the SEM images. We aimed for > 99% spores in our preparations based on light microscopy, consistent with thresholds used in previous studies investigating UV resistance of bacilli^61^. While this standard minimizes confounding effects of impurities, other studies have reported acceptable impurity ranges of 0.5–10% when investigating spore inactivation across various bacilli species^62^. The low level of contamination in our preparations, however, could conflate with the obtained spore values, presumably indicating even lower spore reactivity in the cell assays.

To better understand the differences in bacterial structures potentially involved in microbe-host interactions, we have performed a genome comparison of the tested bacilli strains. We have focused mainly on proteins and carbohydrates on the cell wall of vegetative bacteria and in the outer coat and crust of spores, as they interact with the environment or host organism^32,33^. No clear correlation was found between the presence or absence of protein sequences for exopolymeric substances, or flagella formation, and differences in immunostimulatory capacity between the tested *Bacillus* species or strains. Genes encoding SpsA, SpsE, SpsI, and SpsK, involved in the synthesis of polysaccharides located on the spore surface, were not annotated in *B. licheniformis* AMBY3, whose spores induced the highest activation of the IRF and NF-κB pathways in human monocytes. The presence of these genes, with the exception of *spsA*, was negatively correlated with the induction of NF-κB and IRF pathways based on Pearson correlation, suggesting a role in immune regulation or evasion (Fig. 4B). These carbohydrates are likely the initial point of contact in cell-to-host or cell-to-surface interactions^41–43^, however, exopolysaccharides could also shield other MAMPs on the spore surface from PRR interactions, as shown in the model probiotic *L. rhamnosus* GG^64^. Although our experimental setup cannot establish a definitive link, we hypothesize that polysaccharides may be associated with a lower immune induction capacity in spores. This hypothesis should be validated by measuring bacterial surface hydrophobicity or engineering bacilli mutants in polysaccharide-related genes.

We have also uncovered a difference in presence of genes encoding for SscA and spore coat proteins (Cot) CotB, CotC, CotG and CotQ between the tested *Bacillus* strains, and their immunostimulatory capacity has not yet been discussed in literature^65,66^. Presence of the *cotB*, *cotG* and *sscA* genes was found to be negatively correlated with TLR2/6 activation and induction of IRF and NF-κB pathways (Fig. 4B). A trend for a relative higher IRF and NF-κB induction was observed by *B. velezensis* AMBY9 that lacks the *cotQ* sequence compared to *B. velezensis* AMBY1. Furthermore, *B. licheniformis* AMBY3 spores triggered the highest IRF and NF-κB induction in monocytes compared to the other tested strains, while *sscA*, *cotBCGQ* and *spsAEIK* genes encoding spore coat proteins were not annotated in this strain, further highlighting their role in possibly evading immune induction. To our knowledge, these spore-related gene products have not yet been described in literature to reduce immunostimulation by bacilli at the level of TLRs or transcription factors. Overall, it is highly likely that a unique combination of MAMPs per species or strain, determines the immunostimulatory properties of the tested bacteria. Therefore, follow-up studies should be performed with mutants in spore coat-encoding genes and other MAMPs to unravel their role in the observed immunostimulatory capacity of environmental bacilli. Some species used in the study, such as *B. subtilis*, are known to be amenable to knock-out mutations, which could be used to narrow down critical components of the spore surface involved in the immune interactions. However, inactivating molecular determinants of the immune response can also have unexpected secondary effects. Spore component knock-outs may lead to unexpected structural alterations, change of surface properties, or uncovering other MAMPs. For example, inactivation of crust genes in *B. subtilis*, such as *cotVWXYZ* and *cgeAB*, has been shown to lead to broad impacts on the cells, including increased surface hydrophobicity, disruption of the spore PS layer and crust structure, and hindering the attachment of the crust to the rest of the coat^67^. This makes prediction of the narrow immunostimulatory role of specific spore compounds challenging and requires further investigation.

In conclusion, this study shows that environmental *Bacillus* strains and their spores inactivated by heat/UV-C with preservation of surface structures in mind induce the key transcription factors IRF and NF-κB and immune receptor TLR2/6. The type of inactivation method has a significant influence on the immunostimulatory capacity of *Bacillus* strains. Inactivated *Bacillus* spores were generally less immunostimulatory than their vegetative counterparts, and the induction capacity was species- and strain-dependent. This could be explained by the difference in bacterial cell surface composition of surface coat proteins and polysaccharides related to spore formation, such as SpsAEIK and CotBCGQ. Future studies should explore promising strains that emerged from this study, such as *B. velezensis* AMBY1, to validate their immunostimulatory efficacy in vivo.

## Methods

### Bacterial strains and spore production

Bacterial strains used in this study are listed in Table 2. Environmental *Bacillus* strains, which were kindly provided by Living Technologies cvba, were grown on a shaker at 200 rpm at 37 °C in Lysogeny broth (LB) (Sigma Aldrich). *L. rhamnosus* GG was grown statically at 37 °C, 5% CO_2_, in de Man, Rogosa and Sharpe (MRS) broth (Difco). *E. coli* DH5α was grown at 37 °C in LB broth at 200 rpm.

**Table 2:** *Bacillus* species and bacterial controls used in this study and their relevant properties.

Strains	Label	Relevance of other strains of this species	References
*Bacillus* species			
*Bacillus velezensis*	AMBY1 AMBY9	• Feed additive (animals) • Microbial biodegradation and remediation • Production organism for biosurfactants	^13,78^
*Bacillus licheniformis*	AMBY3	• Probiotic in humans • Food and feed additive • Production organism for enzymes, and biosurfactants • Microbial biodegradation and remediation • Microbial-based cleaning products	^3,10,13,79^
*Bacillus subtilis*	AMBY7 AMBY8	• Probiotic in humans and animals • Food and feed additive • Production organism for enzymes, antibiotics, vitamins, biosurfactants, and amino acids • Vaccine preparation • Production of fermented foods • Microbial biodegradation and remediation • Microbial-based cleaning products	^3,10,13,80–82^
*Bacillus pumilus*	AMBY10 AMBY11	• Production organism for enzymes • Microbial biodegradation and remediation • Microbial-based cleaning products	^3,10,83^
**Controls**			
*Escherichia coli*	DH5α	• Non-pathogenic • Normal flora of the human intestine	^84,85^
*Lacticaseibacillus rhamnosus*	GG	• Well-studied probiotic strain with immunomodulating and anti-pathogenic characteristics.	86

To study the difference in immunomodulatory capacity between vegetative and sporulated *Bacillus* species, *Bacillus* spp. were sporulated through nutrient depletion by incubating bacteria for 30 days in LB medium at 37 °C on a rotary shaker KS260B (IKA) at 250 rpm. To remove cell debris and vegetative cells, spores were isolated by centrifugation at 5000 x g for 10 min at 4 °C using a Sigma 4-16KS centrifuge and washed twice with distilled water (dH_2_O), and finally resuspended in 50% ethanol. After incubation at 28 °C for 2 h at 250 rpm^68^, the spore suspension was washed twice with dH_2_O and further purified using sucrose (50%) density gradient centrifugation (5000 x g, 5 min, 4 °C) to remove the cell debris and remaining vegetative cells, as described by Weldy et al.^69^. The spore pellet was washed 5 times, each time followed by centrifugation (5000 x g, 10 min, 4 °C), and finally resuspended in 2 mL sterile ddH_2_O. The absence of vegetative cells was confirmed by Schaeffer–Fulton stain. The spore suspensions were stored at 4 °C until further use. Final spore concentrations were determined by plating the colony-forming units per mL (CFU/mL) on LB agar plates.

### Inactivation methods

Inactivation requirements were determined using a panel of *Bacillus* strains, as representative for the *Bacillus* species used in the study, which included *B. velezensis* AMBY1, *B. licheniformis* AMBY4, *B. subtilis* AMBY8, and *B. pumilus* AMBY11. Bacterial pellets were obtained by centrifugation at 4000 x g for 10 min and washed in sterile PBS at 2 × 10^6^ CFU/mL. After inactivation with formalin, ethanol, heat and/or UV-C (as described below), the medium was renewed by reducing the supernatant to 100 µL after centrifugation at 4000 x g for 10 min and subsequently adding fresh medium to restore the original volume to compensate for altered cell medium composition. Inactivated cultures were stored at 4 °C and used the next day. Inactivation was confirmed by checking the formation of colonies after plating, and additional UV-C exposure of 1 h was performed on samples which showed growth. The bacterial suspensions were then used for “Co-incubation of vegetative and sporulated *Bacillus* with THP1-Dual™ NF-κB and IRF reporter monocytes”, as described further.

### Formalin and ethanol inactivation

Formalin and ethanol inactivation was based on the methodologies described by Taddese et al.^37^ with adjustments. Formalin inactivation was tested by resuspending the bacterial pellets in a formalin: PBS solutions at 0.5%vv or 1%vv for 1 h, 6 h, or 24 h; in 10%vv for 1 h; or 100%vv for 5–10 min at 20 °C on a shaker at 150 rpm. In case of ethanol inactivation, pellets were resuspended in 2 mL of 10%vv or 100%vv ethanol: PBS, followed by incubation on a shaker at 150 rpm at 20 °C for 15 or 5 min, respectively. After incubation, formalin and ethanol were discarded by centrifuging the suspension at 4000 x g for 5 min and resuspending the pellet in corresponding media for human cell experiments.

### UV-C and heat inactivation

UV inactivation was tested based on a method described by Spacova et al.^23^. Bacteria were divided into 2 mL Eppendorf tubes containing 1mL of culture and placed in the center of a UV Clave™ Ultraviolet Chamber (Benchmark Scientific, 254 nm, 600 mJ/cm2 per 20 min cycle) for 1, 2, 3, or 4 h consisting of consecutive 20-min rounds of UV-C irradiation followed by intermediate vortexing. In case of heat inactivation, 2mL Eppendorf tubes containing 1mL of culture were heat-treated using a dry heat block (Starlab Dry Bath Heating System) at 50 °C, 60 °C, 70 °C, 80 °C for 15, 20, 30, and 40 min. Hereafter, samples were immediately placed on ice^37^.

For a combination of heat and UV-C irradiation, bacterial cultures were resuspended in the corresponding media for human cell experiments and inactivated for 20–30 min at 60 °C, 70 °C, or 80 °C, followed by UV-C exposure for 2 h, 2.5–3 h.

### Scanning electron microscopy (SEM) visualization

*B. pumilus* AMBY11 was visualized using scanning electron microscopy (SEM) to observe the morphological changes on the bacterial surfaces after 10% formalin or heat/UV (20 min at 60 °C, followed by 3 h UV-C) inactivation, as described by Wuyts et al.^70^. Briefly, *B. pumilus* AMBY11 was inactivated as described above and washed twice with PBS. Bacteria were spotted on a gold-coated membrane (appr. 10^8^ CFU per membrane) and fixed with 2.5% (m/v) glutaraldehyde in 0.1 M sodium cacodylate buffer (2.5% glutaraldehyde, 0.1 M sodium cacodylate, 0.05% CaCl_2_.2H_2_O at pH 7.4) by gentle shaking for 1 h at RT, followed by overnight fixation at 4 °C. The membrane was washed afterwards (3 times, 20 min) and left overnight in cacodylate buffer containing 7.5% (m/v) saccharose. Subsequently, bacteria were dehydrated at RT in a series of ethanol solutions with increasing concentrations (50% (10 min), 70% (15 min), 90% (15 min), 95 % (15 min), and 100% (3 × 30 min)). Then, the bacteria were critical point dried in a Leica EM CPD030 (Leica Microsystems Belgium, Diegem, Belgium). The membranes were mounted on a stub and coated with 10 nm of carbon in a Leica EM Ace 600 coater (Leica Microsystems Belgium). SEM imaging was performed with a Quanta FEG250 SEM system (Thermo Fisher, Asse, Belgium; joint infrastructure of the Electron Microscopy for Material Science (EMAT) and CBH research groups, University of Antwerp).

### Measurement of nucleic acid release

Inactivated spore suspensions were centrifuged at 8000 x g for 15 min at 4 °C. Absorbance measurements at 260 nm and 280 nm were performed on the supernatant using a UV/Vis spectrophotometer (Nanodrop One, Thermo Scientific). Sterile dH_2_O served as a blank control, while the untreated group functioned as a negative control.

### Co-incubation of *Bacillus* spp. with THP1-Dual™ NF-κB and IRF reporter monocytes

Induction of NF-κB and IRF pathways by vegetative and sporulated bacteria was determined by using THP1-Dual™ human reporter cells (InvivoGen, USA), as described by Spacova et al.^23^. Briefly, THP1-Dual™ monocytes (InvivoGen) were cultured in Roswell Park Memorial Institute (RPMI) 1640 medium supplemented with 2 mM L-glutamine (Gibco, Life Technologies), 25 mM HEPES (Gibco, Life Technologies), 10% (v/v) heat-inactivated fetal bovine serum (FBS, Gibco, Life Technologies), Normocin (100 µg/mL, InvivoGen) and PenStrep (100 µg/mL, Gibco, Life Technologies) at 37 °C in a humidified incubator with 5% CO_2_. THP1-Dual™ cells were seeded on the day of the co-incubation experiment in flat-bottom 96-well plates (VWR) at a concentration of 1 × 10^6^ cells/mL together with 1 × 10^6^ UV-inactivated bacteria. Activation of NF-κB and IRF pathways in THP1-Dual™ cells was measured by secreted alkaline phosphatase (SEAP) and luciferase production, respectively. NF-κB activation was quantified by measuring SEAP activity at OD405 with the Synergy HTX Plate Reader (BioTek) after combining 50 µL supernatant from each well with 100 µL para-nitrophenyl phosphate (pNPP) substrate buffer (1.5 mg/mL pNPP, 100 mM Tris-HCl, 100 mM NaCl and 5 mM MgCl_2_). IRF activation was determined by luciferase activity using the Synergy HTX Plate Reader with a luminescence measurement after the addition of the QUANTI-Luc™ (InvivoGen) buffer. Lipopolysaccharide (LPS) from *E. coli* (Sigma) was added at 10 ng/ml as positive control.

### Co-incubation of *Bacillus* spp. with HEK-Blue™ hTLR4 and HEK-Blue™ hTLR2-hTLR6 cells

HEK-Blue™ hTLR4 and HEK-Blue™ hTLR2-hTLR6 cells were used to measure activation of human TLR2/6 and TLR4 (InvivoGen, San Diego, CA, USA) after exposure to the bacterial strains through the production of SEAP, as described by De Boeck et al.^71^. Briefly, HEK-Blue™ cells were cultured in Dulbecco’s Modified Eagle Medium (DMEM) with 4.5 g/L glucose (Gibco, Life Technologies) and 2 mM L-glutamine (Gibco, Life Technologies), 10% (v/v) FBS, normocin (100 µg/mL, InvivoGen), PenStrep (100 µg/mL, Gibco, Life Technologies) at 37 °C in a humidified incubator containing 5% CO_2_. One day prior to the co-incubation experiment, HEK-Blue™ cells were seeded into flat-bottom 96 well plates (VWR) at 1 × 10^6^ live cells/mL. After 24 h, inactivated bacteria at a concentration of 2 × 10^6^ CFU/mL were added to the HEK-Blue™ hTLR4 and HEK-Blue™ hTLR2-hTLR6 cells. *Escherichia coli* LPS (O55:B5) was used as a positive control for HEK-Blue™ hTLR4, whereas synthetic diacylated lipopeptide Pam2CSK4 (InvivoGen) was used for HEK-Blue™ hTLR2-hTLR6 cells. Activation of TLR4 and TLR2/6 was quantified by combining 50 µL of supernatant from each well with 100 µL pNPP substrate buffer. Subsequently, the activity of SEAP was determined at an absorbance of 405 nm using the Synergy HTX plate reader.

### Cell viability MTT assay

Cell viability of HEK-Blue™ cells after exposure to bacteria was determined through an MTT cytotoxicity assay. Exposed cells were washed with PBS and 100 µL MTT solution [1 mg/mL 3-[4,5-dimethylthiazol-2-yl]−2,5 diphenyltetrazolium bromide (MTT) in DMEM] was added. After incubation for 3 h, the supernatant was removed and the formed formazan was dissolved in 200 µL dimethyl sulfoxide (DMSO). Cell viability was determined by measuring OD at 550 nm using the Synergy HTX plate reader.

### Genome sequencing, assembly, and in silico analysis

For whole genome sequencing, bacilli were grown overnight in LB-medium and bacterial DNA was extracted using a phenol/chloroform extraction protocol, after which the DNA was purified by ethanol precipitation^72^. After extraction, the DNA concentration and purity were determined by spectrophotometry using a Qubit life (Invitrogen). Whole genome sequencing was performed after sample preparation with the NexteraXT DNA Sample Preparation Kit (Illumina, United States of America) on the Illumina MiSeq platform (Illumina, United States of America) using 2 × 250 cycles at the Laboratory of Medical Microbiology (University of Antwerp, Belgium). *De novo* assembly of the genomes was performed with SPAdes (v3.12.0). Analysis was then performed using an inhouse whole genome annotation pipeline (https://github.com/LebeerLab/nf-whole-genome-illumina). Quality control and completeness of the genomes were verified by CheckM v1.0.12^74^. Taxonomic classification was based on the average nucleotide identity (ANI) method. Generated FASTA files were screened on spore coat proteins and extracellular structures related to exopolymeric substances (EPS), S-layer, flagella, and pili. This screening was performed using gene names and Protein Data Bank identifiers as provided by references^31,74,75^ analyzed with eggNOG^76^ and PATRIC^77^. Specifically, a more extensive analysis was conducted for spore coat proteins. A local BLAST protein database was created based on Secaira-Morocho et al.^75^ and annotated genomes used in this paper were analyzed against this database using NCBI BLASTp (v2.12.0+). Resulting hits were filtered with a maximum E-value of 0.001 and a minimum bit-score of 40, serving as the limit of detection. Pearson correlation was performed between normalized cell culture read-outs and distinct profiles of genes related to the outer spore layer, crust, and unspecified localization in the spore envelope using the python (v3.10.12) packages pandas (v2.0.3) and scipy (v1.11.1). Normalization of the readout signals was performed using the readout of the positive and negative control. Profiles were created on gene presence/absence across the isolates. P-values were adjusted using the Benjamini-Hochberg multiple testing correction. The correlation was visualized using Seaborn clustermap (v0.13.0).

### Statistical analysis

The assays in the present study were performed in triplicate. Data from in vitro assays were analyzed in GraphPad Prism version 10.0.2. Depending on the normality, which was tested for the data from in vitro assays using the Kolmogorov–Smirnov test, one-way ANOVA with Dunnett’s multiple comparisons test or the Kruskal–Wallis test with Dunn’s multiple comparisons test was performed. A p-value significance threshold of 0.05 was used for all statistical tests.

## Supplementary Information

Below is the link to the electronic supplementary material.

Supplementary Material 1

## Data Availability

The sequencing data for the tested *Bacillus* strains has been deposited into the European Nucleotide Archive (ENA) database under “Environmental Bacillus isolates from soil” project name, with accession number PRJEB87312 (https://www.ebi.ac.uk/ena/browser/view/PRJEB87312). Other datasets generated during and/or analyzed during the current study are available from the corresponding author (Irina.spacova@uantwerpen.be) on reasonable request.
