# Supplementary material for: Immunostimulatory activity of inactivated environmental Bacillus isolates and their endospores
Source: Sci Rep. 2025 Aug 20;15:30604. doi: 10.1038/s41598-025-12833-7 (PMC12368082; doi:10.1038/s41598-025-12833-7)
Supplement: Supplementary file 1 — Supplementary Material 1 [file 41598_2025_12833_MOESM1_ESM.pdf]

Supplementary material

Immunostimulatory activity of inactivated environmental *Bacillus* isolates and their endospores

Max Dekeukeleire[1,2], Dieter Vandenheuvel[1], Tippapron Khondee[1], Lize Delanghe[1], Tim Van Rillaer[1], Sofie Thys[3,4], Jean-Pierre Timmermans[3,4], Sarah Lebeer[1], Irina Spacova[1]*

[1] Laboratory of Applied Microbiology and Biotechnology (LAMB), Department of Bioscience Engineering, University of Antwerp, Antwerp, Belgium.

[2] Research group EnVOC (Environmental Organic Chemistry and Technology), Department of Green Chemistry and Technology, Faculty of Bioscience Engineering, Ghent University, Ghent, Belgium.

[3] Laboratory of Cell Biology and Histology (CHB), Department of Pharmaceutical, Biomedical and Veterinary Sciences, University of Antwerp, Wilrijk, Belgium.

[4] Antwerp Centre for Advanced Microscopy (ACAM), University of Antwerp, Wilrijk, Belgium.

*Correspondence: Irina.spacova@uantwerpen.be

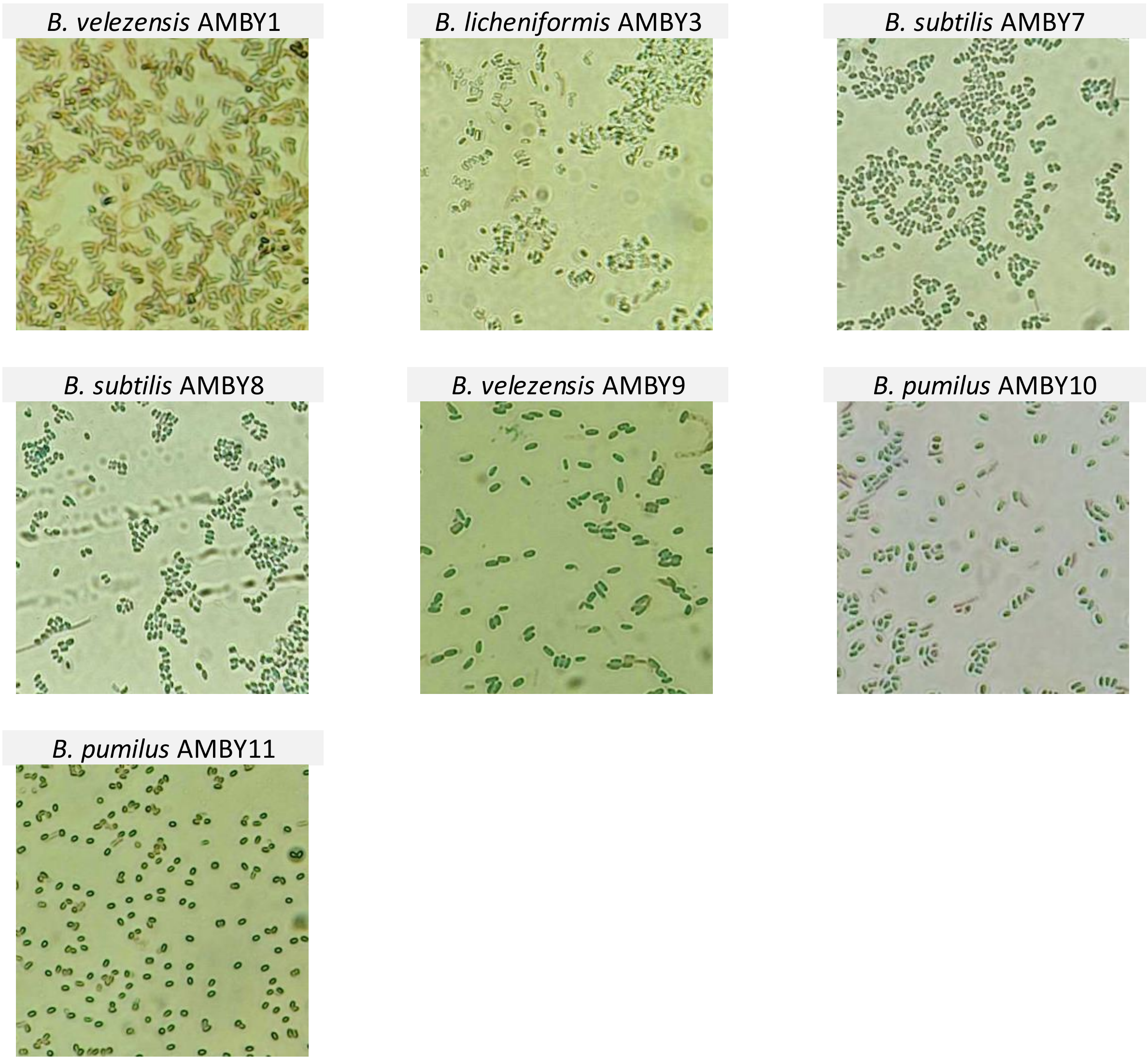

Figure S 1: Schaeffer–Fulton staining of purified spores. Spores are stained with malachite green stain and appear light blue/green; vegetative cells are stained with safranin and appear pink/red. Percentage of vegetative cells in the purified spore solutions in these samples were low and quantified as 0% (AMBY3, AMBY11), 0.5% (AMBY7), 0.4% (AMBY8), 1.3% (AMBY9), 4.4% (AMBY10) and 4.5% (AMBY1). Spore solutions of strains such as AMBY1 were purified again after taking these images.

Table S1: *Bacillus* colony count after inactivation by heat, UV-C, ethanol and formalin. Heat inactivation was tested for 50-100°C for 15 to 40 minutes. UV-C radiation was tested for 1h to 4h. Ethanol inactivation was tested by exposure to 100% ethanol for 5 min or 10% for 15 min. Formalin inactivation was investigated for 5min to 24h, with formalin concentrations ranging from 0.5% to 100%.

Heat inactivation																				
Duration (min)	***Bacillus velezensis* AMBY1**					***Bacillus licheniformis* AMBY4**					***Bacillus subtilis* AMBY8**					***Bacillus pumilus* AMBY11**				
	**50°C**	**60°C**	**70°C**	**80°C**	**100°C**	**50°C**	**60°C**	**70°C**	**80°C**	**100°C**	**50°C**	**60°C**	**70°C**	**80°C**	**100°C**	**50°C**	**60°C**	**70°C**	**80°C**	**100°C**
15	>LOD	51	30	35.7	0	>LOD	23.7	1.7	1.3	0	>LOD	73	66	55.7	0	>LOD	1.3	1	2.3	0
20	>LOD	49	31	32.7	0	>LOD	5.3	1.7	1.3	0	>LOD	74	73	38	0	>LOD	1.3	2	0.3	0
30	>LOD	42.7	21	30.3	0	>LOD	6	1.7	0.7	0	>LOD	69	74	34	0	>LOD	1.3	1.7	0.3	0
40	>LOD	52.6	36.3	22.7	0	>LOD	25	0.7	1.3	0	>LOD	69	50	29	0	>LOD	0.7	0.7	0	0

UV-C inactivation				
**Bacteria**	**1h**	**2h**	**3h**	**4h**
***Bacillus velezensis* AMBY1**	24.0	1.0	0.0	0.0
***Bacillus licheniformis* AMBY4**	>LOD	>LOD	>LOD	>LOD
***Bacillus subtilis* AMBY8**	>LOD	2.0	0.0	0.0
***Bacillus pumilus* AMBY11**	>LOD	71.5	3.0	0.5

Ethanol inactivation		
**Bacteria**	**5 min, 100%**	**15 min, 10%**
***Bacillus velezensis* AMBY1**	12.3	12.3
***Bacillus licheniformis* AMBY4**	2.0	0.3
***Bacillus subtilis* AMBY8**	>LOD	>LOD
***E. coli* DH5α**	0.0	0.0

Formalin inactivation					
	**5 min**	**10 min**	**1h**	**6h**	**24h**
0.5%	-	-	>LOD	>LOD	>LOD
1%	-	-	>LOD	>LOD	>LOD
10%	-	-	0.0	-	-
100%	0.0	0.0	-	-	-

Table S2: Effects of formalin and heat/UV-C treatments on the release of nucleic acid from *Bacillus* spores determined using a Nanodrop One. Background corrected with dH_2O.

**Bacteria**	**Inactivation**	**ng/µL**	**A260/280**	**A260/230**
Bacillus velezensis AMBY9	Control	0.4	-1.05	-0.22
	Formalin	0.6	-1.4	-0.08
	Heat/UV-C	-0.4	0.41	0.06
Bacillus pumilus AMBY11	Control	-1.4	0.86	0.04
	Formalin	-1.5	0.95	0.04
	Heat/UV-C	2.1	1.67	0.06

Table S3: Overview of annotated genes of bacilli extracellular structures: EPS [1], S-layer [2], and flagellum [3,4] (green: detected, grey: no hits).

	EPS	*B. pumilus* AMBY11	*B. pumilus* AMBY10	*B. velezensis* AMBY1	*B. velezensis* AMBY9	*B. subtilis* AMBY8	*B. subtilis* AMBY7	*B. licheniformis* AMBY3	Exopolymeric Substance
**Structural**	*epsA*	grey	grey	grey	grey	grey	grey	grey	Uncharacterized exopolysaccharide
	epsB	grey	grey	grey	grey	grey	grey	grey	
	epsC	grey	grey	grey	grey	grey	grey	grey	
	epsD	green	green	green	green	green	green	green	
	epsE	green	green	green	green	green	green	green	
	epsF	green	green	green	green	green	green	green	
	epsG	green	green	green	green	green	green	green	
	epsH	green	green	green	green	green	green	green	
	epsI	green	green	green	green	green	green	green	
	epsJ	green	green	green	green	green	green	green	
	epsK	green	green	green	green	green	green	green	
	epsL	green	green	green	green	green	green	green	
	epsM	green	green	green	green	green	green	green	
	epsN	green	green	green	green	green	green	green	
	epsO	grey	grey	green	green	green	green	green	
	sacB	grey	grey	green	green	green	green	green	Levan
	sacC	grey	grey	grey	grey	green	green	green	
	yhxB	grey	grey	grey	grey	grey	grey	grey	Uncharacterized exopolysaccharide
**Sorptive**	*pgsA*	grey	grey	grey	grey	grey	grey	grey	Poly(γ-glutamic acid)
	pgsB	green	green	green	green	green	green	green	
	pgsC	grey	grey	grey	grey	grey	grey	grey	
**Surface active**	*srfAA*	green	green	green	green	green	green	green	Surfactin
	srfAB	green	green	green	green	green	green	green	
	srfAC	green	green	green	green	green	green	green	
	srfAD	green	green	green	green	green	green	green	
	fenF	grey	grey	green	green	grey	grey	grey	Mycosubtilin
	mycA	grey	grey	grey	grey	grey	grey	grey	
	mycB	grey	grey	grey	grey	grey	grey	grey	
	mycC	grey	grey	grey	grey	grey	grey	grey	
	bamA	grey	grey	grey	grey	grey	grey	grey	Bacillomycin
	bamB	green	green	grey	grey	grey	grey	grey	
	bamC	grey	grey	grey	grey	grey	grey	grey	
	bamD	grey	grey	grey	grey	grey	grey	grey	
	ituA	grey	grey	grey	grey	grey	grey	grey	Iturin A
	ituB	grey	grey	grey	grey	grey	grey	grey	
	ituC	grey	grey	grey	grey	grey	grey	grey	
	ituD	grey	grey	grey	grey	grey	grey	grey	
	fenA	grey	grey	grey	grey	grey	grey	grey	Fengycin
	fenB	grey	grey	grey	grey	grey	grey	grey	
	fenC	grey	grey	grey	grey	grey	grey	grey	
	fenD	grey	grey	grey	grey	grey	grey	grey	
	fenE	grey	grey	grey	grey	grey	grey	grey	
**EPS enzymes**	*xynA*	green	green	green	green	green	green	grey	Xylanases
	xynC	green	green	green	green	green	green	grey	
	pel	grey	grey	green	green	green	green	grey	Pectate Lyase
	sfp	green	green	green	green	green	green	green	Phosphopantetheinyl Transferase
	aprE	green	green	grey	grey	green	green	grey	Subtilisin
	nprE	grey	grey	grey	grey	green	green	grey	Neutral metallo protease E
	bpf	grey	grey	grey	grey	grey	grey	grey	Bacillopeptidase F
	mpr	grey	grey	green	green	green	green	grey	Mpr Protease
	epr	green	green	green	green	green	green	green	Epr Protease
	nprB	grey	grey	grey	grey	green	green	grey	NprB
	vpr	green	green	green	green	green	green	green	Vpr
	swrAA	grey	grey	grey	grey	grey	grey	grey	SwrAA
	tasA	green	green	green	green	green	green	green	TasA protein

	Surface structure	*B. pumilus* AMBY11	*B. pumilus* AMBY10	*B. velezensis* AMBY1	*B. velezensis* AMBY9	*B. subtilis* AMBY8	*B. subtilis* AMBY7	*B. licheniformis* AMBY3	Product
**S-Layer**	*aeg*	grey	grey	grey	grey	grey	grey	grey	aeg protein
	bslC	grey	grey	grey	grey	grey	grey	grey	S-layer protein
	bslF	grey	grey	grey	grey	grey	grey	grey	Putative S-layer protein
	bslM	grey	grey	grey	grey	grey	grey	grey	Putative S-layer protein
	bslR	grey	grey	grey	grey	grey	grey	grey	Autolysin (Cell wall hydrolase)
	csaA	green	green	green	green	green	green	green	pyruvyl transferase
	csaB	grey	grey	grey	grey	grey	grey	grey	pyruvyl transferase
	EA1	grey	grey	grey	grey	grey	grey	grey	S-layer protein EA1
	secA2	green	green	green	green	green	green	green	SecA2
	slaQ	grey	grey	grey	grey	grey	grey	grey	SlaQ
	slaP	grey	grey	grey	grey	grey	grey	grey	SlaP
	sap	grey	grey	grey	grey	grey	grey	grey	Crystal protein
**Flagel**	*fliC*	green	green	green	green	green	green	green	filament
	fliD	green	green	green	green	green	green	green	filament cap
	Flik	green	green	green	green	green	green	green	Flagellar hook-length control protein
	flgD	green	green	green	green	green	green	green	Hook cap
	flgE	grey	grey	grey	grey	grey	grey	grey	hook
	FlgF	grey	grey	grey	grey	grey	grey	grey	proximal rod
	flgG	green	green	green	green	green	green	green	distal rod
	flgK	green	green	green	green	green	green	green	Hook-filament junction
	flgL	green	green	green	green	green	green	green	Hook-filament junction

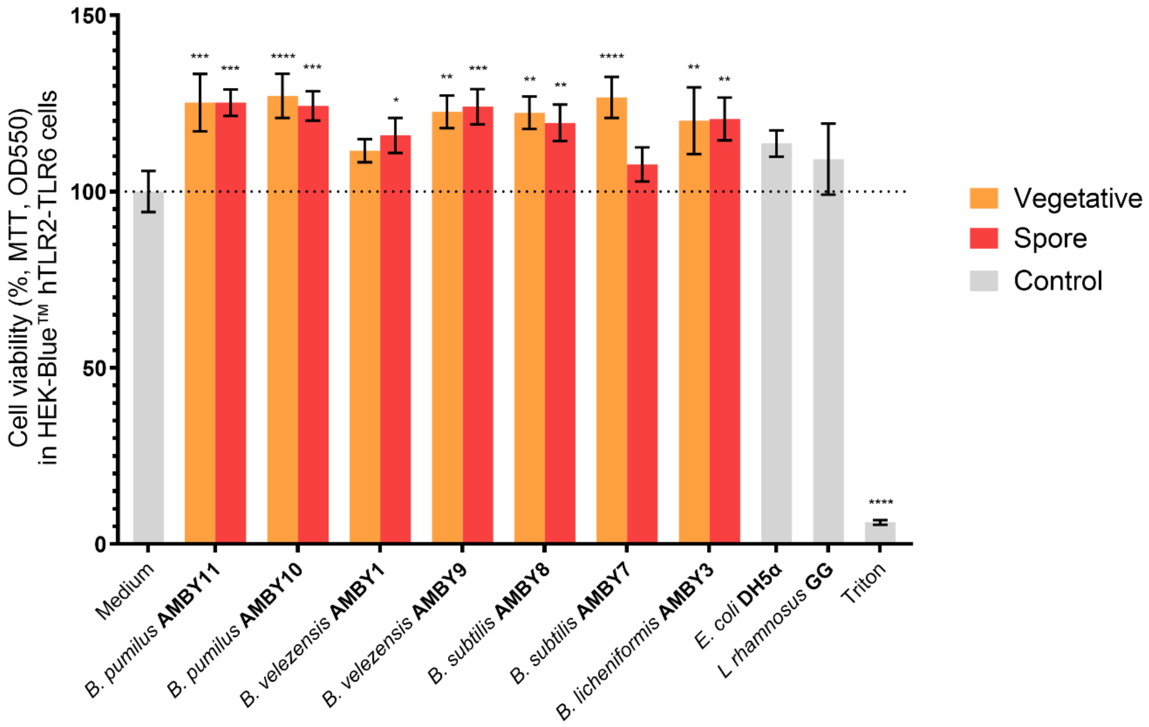

Figure S2: Cell viability of HEK-Blue TLR4 cells after exposure to 10^6 CFU/mL inactivated bacteria for 24 h. *p<0.05, **p<0.01, ***p<0.001, and ****p<0.0001 as determined with One-way ANOVA, followed by Dunett's multiple comparison test compared to the medium condition. Triton serves as a cytotoxic positive control.

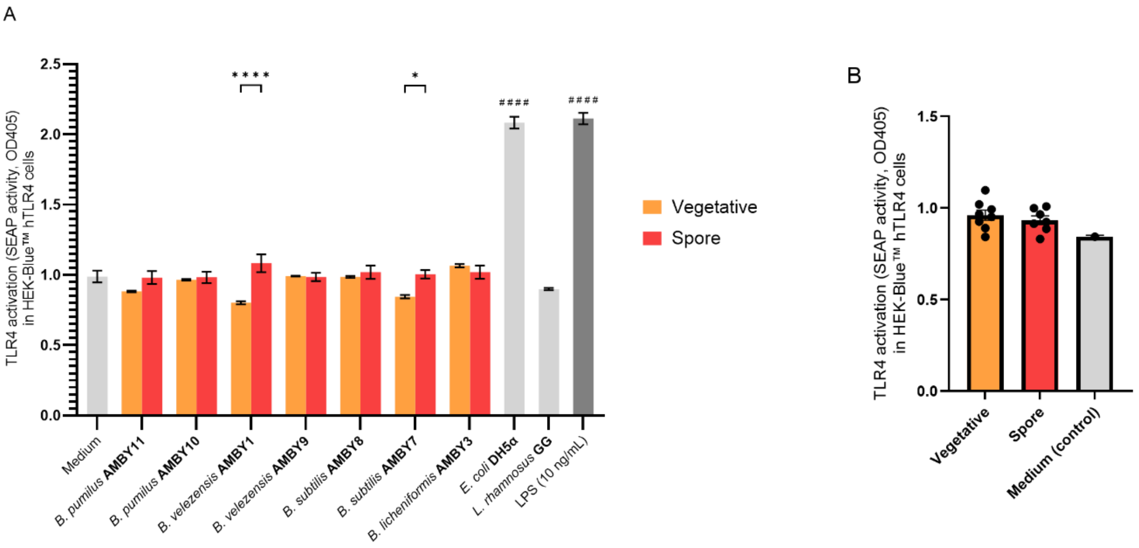

Figure S3: (A) Activation of TLR4 by *Bacillus* spp. and their spores in HEK-Blue™ cells. (B) Average TLR4 activation by spores and vegetative cells. The medium condition represents unexposed cells and serves as a baseline, while lipopolysaccharide (LPS) at 10 ng/ml, *E. coli* DH5α, and *L. rhamnosus* GG, serve as controls. One representative biological replicate is shown. Bars depict means ± SEM. Difference between sporulated and vegetative cells is indicated by *$p<0.05$, **$p<0.01$, ***$p<0.001$, and ****$p<0.0001$ as determined with One-way ANOVA, followed by Dunett's multiple comparison test. Difference compared to the medium control indicated by #$p<0.05$, ##$p<0.01$, ###$p<0.001$, and ####$p<0.0001$.

Table S4: P-values of One-way ANOVA analyses followed by Dunett's multiple comparison test compared between the vegetative or sporulated strains inducing immunoinduction or receptor activation. Difference between sporulated and vegetative cells inducated by *p<0.05, **p<0.01, ***p<0.001, and ****p<0.0001.

**NF-κB**	E. coli	LGG	AMBY11	AMBY10	AMBY1	AMBY9	AMBY8	AMBY7	AMBY3
E. coli **DH5α**		****	0.9996	1.000	**	0.133	1.000	0.208	0.98
L. rhamnosus **GG**	****		***	****	****	****	****	****	****
B. pumilus **AMBY11**	****	**		0.9993	***	*	0.994	0.065	0.752
B. pumilus **AMBY10**	****	*	0.972		**	0.144	1.000	0.224	0.982
B. velezensis **AMBY1**	****	0.948	***	**		0.654	*	0.4975	*
B. velezensis **AMBY9**	****	0.977	*	0.307	0.380		0.3903	1.000	0.652
B. subtilis **AMBY8**	****	**	1.000	0.993	***	0.058		0.522	1.000
B. subtilis **AMBY7**	****	***	1.000	0.744	****	**	0.997		0.799
B. licheniformis **AMBY3**	***	****	**	***	****	****	**	*	

**IRF**	E. coli	LGG	AMBY11	AMBY10	AMBY1	AMBY9	AMBY8	AMBY7	AMBY3
E. coli **DH5α**		***	*	0.535	0.763	0.996	0.402	0.102	0.449
L. rhamnosus **GG**	****		0.4039	*	*	**	0.125	0.178	*
B. pumilus **AMBY11**	****	0.927		0.830	0.615	0.187	0.990	1.000	0.892
B. pumilus **AMBY10**	****	1.000	0.817		1.000	0.954	1.000	0.982	1.000
B. velezensis **AMBY1**	****	0.636	0.118	0.778		0.996	0.998	0.893	1.000
B. velezensis **AMBY9**	****	0.999	0.6377	1.000	0.977		0.846	0.420	0.914
B. subtilis **AMBY8**	****	0.998	1.000	0.987	0.343	0.916		1.000	1.000
B. subtilis **AMBY7**	****	1.000	0.9948	1.000	0.564	0.990	1.000		0.993
B. licheniformis **AMBY3**	****	0.093	0.637	0.056	**	*	0.499	0.277	

**TLR2/6**	E. coli	LGG	AMBY11	AMBY10	AMBY1	AMBY9	AMBY8	AMBY7	AMBY3
E. coli **DH5α**		0.703	****	****	****	****	****	****	****
L. rhamnosus **GG**	0.865		****	****	****	****	****	****	****
B. pumilus **AMBY11**	****	****		0.893	***	0.113	0.929	****	**
B. pumilus **AMBY10**	****	****	1.000		**	0.801	1.000	****	0.135
B. velezensis **AMBY1**	****	****	****	****		0.082	**	0.192	0.651
B. velezensis **AMBY9**	****	****	****	****	****		0.741	***	0.926
B. subtilis **AMBY8**	****	****	0.596	0.408	****	****		****	0.110
B. subtilis **AMBY7**	****	****	****	****	1.000	****	****		**
B. licheniformis **AMBY3**	****	****	0.998	0.975	****	****	0.962	****	

**TLR4**	E. coli	LGG	AMBY11	AMBY10	AMBY1	AMBY9	AMBY8	AMBY7	AMBY3
E. coli **DH5α**		****	****	****	****	****	****	****	****
L. rhamnosus **GG**	****		1.000	0.400	0.055	0.082	0.120	0.618	***
B. pumilus **AMBY11**	****	0.925		0.149	0.177	*	*	0.926	****
B. pumilus **AMBY10**	****	0.917	1.000		***	0.99	0.999	*	*
B. velezensis **AMBY1**	****	0.130	0.783	0.798		****	****	0.865	****
B. velezensis **AMBY9**	****	0.902	1.000	1.000	0.820		1.000	**	0.248
B. subtilis **AMBY8**	****	0.6019	1.000	1.000	0.985	1.000		**	0.177
B. subtilis **AMBY7**	****	0.753	1.000	1.000	0.941	1.000	1.000		****
B. licheniformis **AMBY3**	****	0.599	1.000	1.000	0.985	1.000	1.000	1.000	

1 Marvasi, M., Visscher, P. T. & Casillas Martinez, L. Exopolymeric substances (EPS) from Bacillus subtilis : polymers and genes encoding their synthesis. *FEMS Microbiology Letters* **313**, 1-9 (2010). https://doi.org/10.1111/j.1574-6968.2010.02085.x

2 Boutonnet, C. *et al.* Dynamic Profile of S-Layer Proteins Controls Surface Properties of Emetic Bacillus cereus AH187 Strain. *Frontiers in Microbiology* **13** (2022). https://doi.org/10.3389/fmicb.2022.937862

3 Mukherjee, S. & Kearns, D. B. The structure and regulation of flagella in Bacillus subtilis. *Annu Rev Genet* **48**, 319-340 (2014). https://doi.org/10.1146/annurev-genet-120213-092406

4 Hajam, I. A., Dar, P. A., Shahnawaz, I., Jaume, J. C. & Lee, J. H. Bacterial flagellin-a potent immunomodulatory agent. *Exp Mol Med* **49**, e373 (2017). https://doi.org/10.1038/emm.2017.172
